# What did we learn about tocilizumab use against COVID-19? A single-center observational study from an intensive care unit in Serbia

**DOI:** 10.3389/fmed.2023.1253135

**Published:** 2023-11-14

**Authors:** Tatjana Adzic-Vukicevic, Dejan Markovic, Aleksandar Reljic, Voin Brkovic

**Affiliations:** ^1^Clinic of Pulmonology, University Clinical Center of Serbia, Belgrade, Serbia; ^2^Faculty of Medicine, University of Belgrade, Belgrade, Serbia; ^3^Clinic for Anesthesiology, University Clinical Center of Serbia, Belgrade, Serbia; ^4^Clinic for Nephrology, University Clinical Center of Serbia, Belgrade, Serbia; ^5^Covid Hospital Batajnica, University Clinical Center of Serbia, Belgrade, Serbia

**Keywords:** COVID-19, tocilizumab, superinfections, outcome, complications

## Abstract

**Background:**

Selection of effective and safe therapy for management of patients with coronavirus disease is challenging. Tocilizumab (TZB) has emerged as a potential treatment option for COVID-19. Several aspects regarding Tocilizumab treatment remain uncertain, such as the optimal timing for its administration and the safety profile, including the potential risk of infections. The aim of the study is to present the clinical characteristics of patients with COVID-19 following the application of Tocilizumab.

**Methods:**

This is a retrospective analysis of 121 patients with severe forms of COVID-19 previously treated with Tocilizumab was conducted. All patients were admitted to intensive care units (ICUs).

**Results:**

Of 121 patients, the majority were men 72 (59.5%) with a median age at presentation of 65 ± 13 years. Only 9 (7.43%) patients were without comorbidities, while the other 112 (92.55%) had two or more comorbidities. Almost all of the 120 patients (99.2%) needed oxygen therapy, such as nasal cannulas in 110 (90.9%) patients, high flow nasal catheter (HFNC) in 4 (3.3%) patients, and continuous positive airway pressure (CPAP) in 5 (4.1%) patients while 1 patient was intubated at the time of hospital admission. The average time from Tocilizumab application to admission to the ICU was 3 days. During clinical deterioration, almost half 57 (47.1%) of the patients were intubated, and 52 (82.5%) of these intubated patients (*p* < 0.001) had lethal outcomes. The most significant predictors for a lethal outcome according to multivariate analysis were diabetes mellitus (*p* < 0.001) followed by a subsequent elevation in C-reactive protein levels (CRP; *p* < 0.002) and ferritin (*p* < 0.013) after Tocilizumab application. Bloodstream infections were found in 20 (16.5%) patients, most frequently with Gram-negative pathogens like *Acinetobacter* spp. as in 12 (18.6%) patients, *Klebsiella* spp. in 6 (8%) patients, and *Pseudomonas* spp. in 2 (3.2%) patients. Urine culture isolates were found in 9 (7.43%) patients, with *Candida* spp. being most frequently isolated in 7 (5.8%) patients, followed by *Klebsiella* spp. and *Pseudomonas* spp. in 1 patient each (0.8%). Significantly lower survival was seen in patients with proven infection.

**Conclusion:**

The benefit of tocilizumab was not found in our study. The high mortality rate among intubated patients after Tocilizumab use suggests appropriate patient selection and monitoring and emphasizes the risk of superinfections. Diabetes mellitus, increased levels of CRP, and ferritin were identified as the most significant predictors of poor outcomes in contrast to increased levels of IL-6.

## Introduction

For more than 3 years, the whole world struggled with severe acute respiratory syndrome coronavirus 2 (SARS-CoV-2). By the end of May 2023, at the pandemic’s end, several questions were still without answers, and one of the most important was the selection of an effective and safe treatment for patients with COVID-19 (1). Approximately 15% of COVID-19 patients develop severe disease, while around 5% progress to a critical condition (2). In critically ill COVID-19 patients, cardiovascular collapse, followed by multiorgan dysfunction and shock, could be found as causes of death. Severe forms of SARS-CoV-2 infection are characterized by excessive production of pro-inflammatory cytokines, leading to a phenomenon known as a “cytokine storm” (3). There is a significant increase in the level of cytokines such as interleukin-6 (IL-6), interleukin-2 (IL-2), ferritin, fibrinogen, and lymphocytes. Tocilizumab (TZB) represents a recombinant humanized monoclonal IL-6 receptor antibody of the IgG1 subtype (4). One of the advantages of tocilizumab is its prolonged half-life and irreversible binding to IL-6 receptors, including both the soluble and the membrane-bound forms (5). Likewise, the beneficial role of TZB has been shown in some inflammatory diseases such as rheumatoid arthritis, systemic juvenile idiopathic arthritis, Castleman disease, Crohnʼs disease, and cytokine release syndrome (CRS) induced by chimeric antigen T-cell (CAR-T) immunotherapy (6). Serbian national protocol is supported by the Ministry of Health and contains 13 revisions, including the use of TZB in the therapy of COVID-19 patients with moderate disease and a gradual escalation in their requirement for oxygen support, including the need for mechanical ventilation (7). The use of TZB in critically ill COVID-19 patients is still controversial because of the reported proof that TZB reduced the risk of mechanical ventilation, although it does not have a substantial impact on mortality rate (8, 9). The aim of this study is to present the clinical characteristics of patients with COVID-19 following the application of Tocilizumab.

## Materials and methods

This is an observational retrospective study of 7,949 consecutive patients older than 18 years, treated in intensive care units (ICU; total six, with capacity of 120 beds) in the largest Covid hospital in Europe, Batajnica, Belgrade, from 4 December 2020 to 1 June 2021. Among 7,949 patients, 121 (1.52%) were treated with TZB and, because of clinical deterioration, transferred to ICU. All included patients received TZB previously in second-level hospitals or temporary Covid hospitals.

The inclusion criteria for TZB administration in prior hospitals are unknown.

The criteria for admission to the intensive care unit (ICU) included severe respiratory failure necessitating invasive or non-invasive mechanical ventilation (MV) followed by a deterioration in respiratory function, radiological progression, and positive reverse transcriptase-polymerase chain reaction (RT-PCR) results from nasopharyngeal swabs for SARS-CoV-2. Data regarding the patient’s medical history, vital signs, oxygen saturation, blood chemistry parameters, microbiological analyses, radiological findings (including chest X-ray and computed tomography), treatment regimen, and outcome were recorded for each individual. A CT score, or Computed Tomography score, is a numerical assessment used in medical imaging to quantify and describe findings in CT scans, helping the assessment of the severity or extent of abnormalities in the scanned area. Blood chemistry variables were initially recorded upon presentation then maximum values achieved during hospitalization, as well as variables at discharge from the hospital or time to death. Non-invasive ventilation included continuous positive airway pressure (CPAP), full face masks, and Helmet. Patients who were on high-flow oxygen therapy with a flow rate of 70 L/min receiving a 100% fraction of inspired oxygen (FiO_2_) and who had a partial pressure of oxygen (PO2) below 8 kPa and oxygen saturation below 90%, which is considered as severe respiratory insufficiency, were also transferred to the intensive care unit (ICU).

The treatment of all patients was conducted in accordance with a national protocol for severe forms of COVID-19 disease, including corticosteroid therapy (methylprednisolone up to 2–3 mg per kg), followed by low medium heparin weight (LMHW), gastro-protective, antibiotic, or antifungal therapy based on microbiological or laboratory findings.

### Statistical analysis

The normal distribution of continuous variables was tested using the Kolmogorov–Smirnov test, and variables are presented as mean ± SD or median (interquartile range) as appropriate. Differences in continuous variables between the group of survivors and non-survivors were assessed by Student’s *t* test for normal distributed variables or Mann–Whitney U test for non-normal distributed variables. Categorical variables are presented as counts and percentages and the Chi-square or Fisher’s exact tests were used to analyze differences between analyzed groups. Univariate Cox proportional-hazards regression analyses were used to identify predictors for in-hospital mortality. Variables that show a significant predictive value with a *p*-value of less than 0.1 were included in a multivariate Cox model using a forward stepwise (likelihood ratio) method of entry. Kaplan–Meier survival curves were used to illustrate differences in survival between groups. Statistical significance was assessed by the Log-rank test.

Statistical analyses were performed using the statistical package for social sciences, version 28 (SPSS, Chicago, Ill). Statistical significance was defined as *p* < 0.05.

## Results

**Figure 1:**
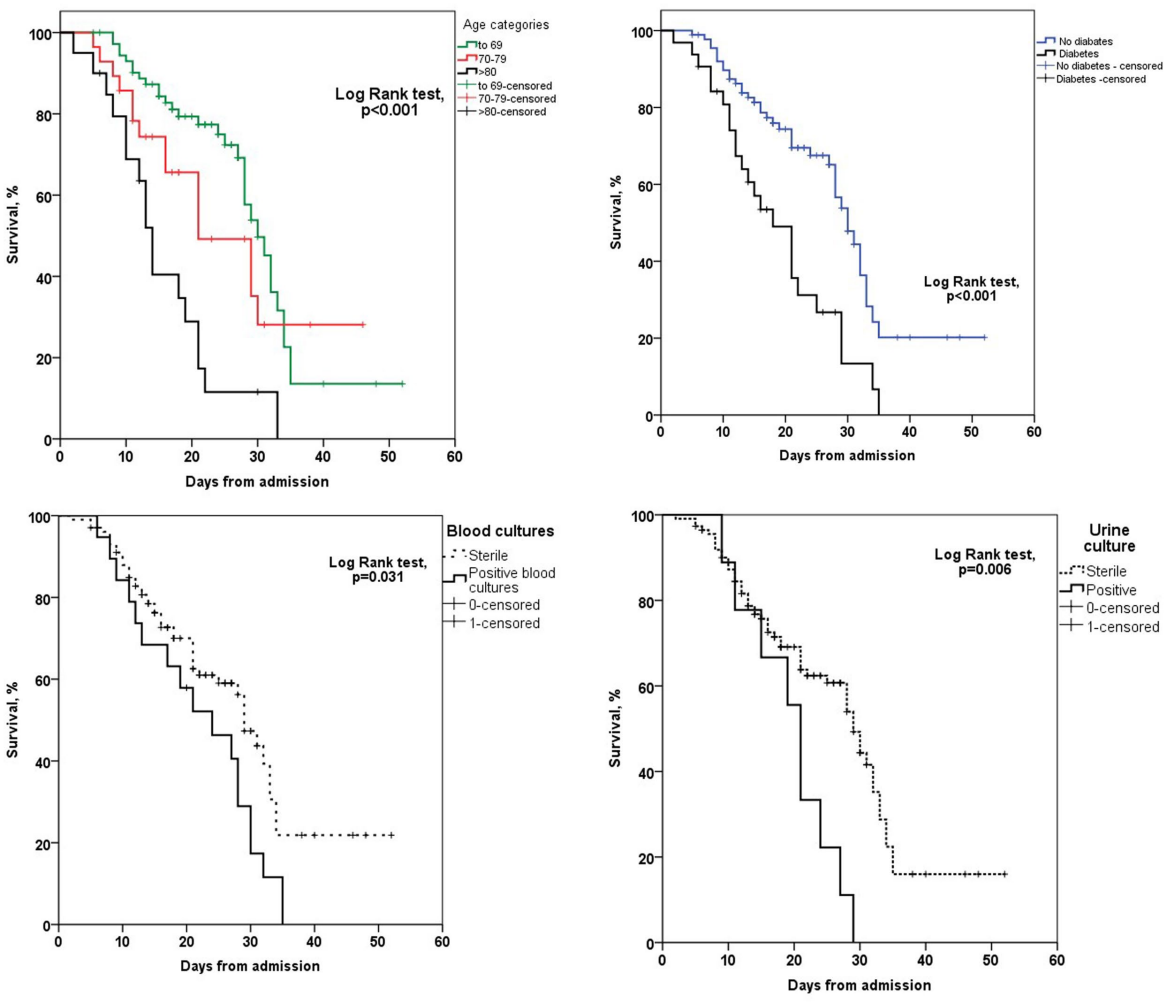
Kaplan–Meier curves survival analysis according to the age category, diabetes status, urine and blood culture.

Out of a total of 7,949 hospitalized patients from December 4, 2020, to June 1, 2021, a cohort of 121 patients was included in the analysis. The median duration from the administration of tocilizumab to admission to the intensive care unit (ICU) was 3 days (IQR 2–4). Additionally, the median duration from the onset of symptoms to ICU admission was 5 days (IQR 3–5.5). The collected data were divided into two groups based on whether patients were survivors or non-survivors. Patient characteristics are shown in Table 1. The majority of patients, 72 patients (59.5%), were men. The mean age at presentation was 65 ± 13 years, with 73 (60.3%) aged fewer than 70 years, 28 (23.1%) aged between 70 and 79 years, and 20 (16.5%) aged older than 80 years. In addition, 9 patients (7.43%) presented without comorbidities, 18 (14.87%) patients had one comorbidity, and 94 (77.68%) had two or more comorbidities. Among the primary non-malignant comorbidities, the most commonly observed conditions were arterial hypertension, present in 70 (57.9%) patients, followed by diabetes mellitus in 32 patients (26.4%) and chronic obstructive pulmonary disease (COPD) in 15 patients (12.4%). Hematological malignancies were most common among malignant diseases (in 4 (3.3%) patients). Most of the patients in our study exhibited typical clinical symptoms, including loss of smell (116, 95.9%) and loss of taste (115, 95%) along with elevated body temperature. Clinical and laboratory findings at the time of hospital admission are presented in Table 2. Antivirals (Favipiravir) were applied in 10 (8.3%) patients markedly in those with a later lethal outcome (*p* < 0.012). The requirement for oxygen therapy was determined based on the oxygen saturation level measured using pulse oximetry. Almost all the patients 120 (99.2%) needed oxygen therapy. At the time of hospital admission, oxygen was delivered using nasal cannulas in 110 (90.9%) patients, high flow nasal catheter (HFNC) in 4 (3.3%) patients, and continuous positive airway pressure (CPAP) in 5 (4.1%) patients, while 1 patient was intubated. The average CT severity score was 14 ± 5.9. All patients received the best basic and supportive care and anti-COVID treatment in accordance with the national protocol, including antibiotics, antimycotics, anticoagulation, and corticosteroid therapy. The average stay in ICU was 6 days, while significant time spent on MV was seen in non-survivors (*p* < 0.001). During clinical deterioration, almost half of the patients were intubated 57 (47.1%), namely those who died later 52 (82.5%; *p* < 0.001). Overall, in cured patients, during time of deterioration, nasal cannulas, HFNC, and CPAP were most applied (*p* < 0.001). Among intubated patients, 52 (82.5%) had lethal outcomes, and only 5 (8.6%) survived. The following potential laboratory prognostic parameters were evaluated: white blood cells (WBC), C-reactive protein (CRP), IL-6, ferritin, and lactate dehydrogenase (LDH.). Regarding the analysis of chest X-ray after TZB administration, in the majority of patients 85 (70.4%)radiographic progression was noticed, improvement was achieved in 19 (15.7%), and chest X-rays without changes were noticed in 17 (13.9%). The most significant predictor for lethal outcome was age (*p* < 0.001, HR 1.045, CI 0.95% 1.022–1.069). Survival according to the age category is shown in Figure 1. Other significant parameters for survival were comorbidities like arterial hypertension (*p* < 0.005, HR 2.162, CI 0.95% 1.261–3.704) and diabetes mellitus (*p* < 0.001, HR 2.46, CI 0.95 1.466–4.083). Among laboratory parameters, CRP after TZB application (*p* < 0.002, HR 1.008, CI 0.95% 1.003–1.013) and ferritin level after TZB application (*p* < 0.001, HR 1.001, CI 0.95% 1.000–1.002) were the most significant (Table 3). Multivariable regression analysis confirmed diabetes mellitus (*p* < 0.001, HR 7.096, CI 0.95% 3.098–16.253) as the strongest predictor for lethal outcome followed by CRP (*p* < 0.022, HR 1.009, CI 0, 95% 1.001–1.016) and ferritin level (*p* < 0.013, HR 1.001, CI 0.95% 1.000–1.002; Table 4). Kaplan–Meier curve number 2 showed influence of diabetes mellitus on survival. Blood stream infections were found in 20 (16.5%). Gram negative pathogens were dominant, *Acinetobacter* spp. in 12 (18.6%), *Klebsiella* spp. in 6 (8%), and *Pseudomonas* spp. in 2(3.2%). Urine culture isolates were found in 9 (7.43) patients, among them *Candida* spp. was most frequently seen in 7 patients, while *Klebsiella* spp. and *Pseudomonas* spp. were seen in 1 patient each. Positive urine culture analyses were significantly found among non-survivors (*p* = 0.03). Significantly lower survival according to positive blood and urine cultures isolates were shown on Kaplan–Meier curves 3 and 4.

**Table 1:** Patients’ characteristics.

	Total *N* = 121	Survival *N* = 58	Non survival *N* = 63	*p*
Men *n* (%)	72 (59.5%)	38 (65.5%)	34 (54.0%)	0.196
Age ± SD	65 ± 13.5 (min–max 30–94)	61 ± 13.1 (min–max 30–94)	70 ± 12.5 (min–max 34–90)	<0.001
Age categories				
<69 years	73 (60.3)	42 (72.4%)	31 (49.2%)	0.003
Between 70–79 years	28 (23.1)	13 (22.4%)	15 (23.8%)	
>80 years	20 (16.5)	3 (5.2%)	17 (27.0%)	
Arterial hypertension, *n* (%)	70 (57.9%)	27 (46.6%)	43 (68.3%)	0.016
Diabetes mellitus, *n* (%)	32 (26.4%)	8 (13.8%)	24 (38.1%)	0.002
Hematological malignancies, *n* (%)	4 (3.3%)	0 (0%)	4 (6.3%)	0.12
HRI, *n* (%)	8 (6.6%)	2 (3.4%)	6 (9.5%)	0.276
COPD, *n* (%)	15 (12.4%)	7 (12.1%)	8 (12.7%)	0.916
Hypothyreosis, *n* (%)	5 (4.1%)	2 (3.4%)	3 (4.8%)	0.717
Previous thrombosis, *n* (%)	8 (6.6%)	3 (5.7%)	5 (7.9%)	0.72

HRI, chronic renal insufficiency; COPD, chronic obstructive pulmonary disease.

**Table 2:** Laboratory and clinical findings at the time of hospital admission.

	Total *N* = 121	Survival *N* = 58	Non survival *N* = 63	*p*
CRP (mg/L) median (IQR)	75.2 (82.03)	71.3 (75.8)	85.8 (91.9)	0.427
WBC (G/L)	8.4 ± 6.64	7.8 ± 6.15	9.1 ± 7.21	0.412
IL-6 (pg/ml)	82.0 (123.15)	82.0 (103.73)	81.45 (131.18)	0.869
Ferritin (ng/ml)	983.5 (1224.0)	1335.8 (1456.1)	767.3 (1104.15)	0.364
LDH (U/l)	447.5 (422.0)	447.5 (369.5)	447.0 (501.75)	0.71
Favipiravir, *n* (%)	10 (8.3%)	1 (1.7%)	9 (14.3%)	0.012
Oxygenotherapy *n* (%)				
Nasal cannulas	110 (90.9%)	55 (94.8%)	55 (87.3%)	0.199
HFNC	4 (3.3%)	0 (0%)	4 (6.3%)	
CPAP	5 (4.1%)	2 (3.4%)	3 (4.8%)	
MV	1 (0.8%)	0 (0.0%)	1 (1.6%)	
Ambiental air	1 (0.8%)	1 (1.7%)	0 (0%)	
Oxygenotherapy at worsening, *n* (%)				
Oxygen mask	21 (17.4%)	20 (34.5%)	1 (1.6%)	<0.001
HFNC	26 (21.5%)	21 (36.2%)	5 (7.9%)	
CPAP	13 (10.7%)	8 (13.8%)	5 (7.9%)	
MV	57 (47.1%)	5 (8.6%)	52 (82.5%)	
Ambiental air	4 (3.3%)	4 (6.9%)	0 (0%)	
Time from symptoms beginning to prior hospital admission (IQR)	5 (4)	5.5 (3)	5 (5)	0.137
Time from hospital admission to TZB application (IQR)	3 (3)	4 (2)	3 (2)	0.472
Days spent in ICU	6 (5)	8 (8)	6 (6)	0.086
Days spent on MV	1 (5)	0 (0)	3 (5)	<0.001
CT severity score	14 ± 5.9	16 (10)	13 (13)	0.855

CRP, C-reactive protein; WBC, White blood cells; IL-6, interleukin-6; LDH, lactate dehydrogenase; HFNC, high flow nasal cannulas; CPAP, continuous positive airway pressure; MV, mechanical ventilation; TZB, tocilizumab; ICU, intensive care unit; MV, mechanical ventilation, CT, computed tomography.

**Table 3:** Univariate regressive model for lethal outcome predictors.

	B	P	HR	CI 0.95%
Age	0.044	<0.001	1.045	1.022–1.069
Arterial hypertension	0.771	0.005	2.162	1.261–3.704
Diabetes mellitus	0.895	0.001	2.460	1.466–4.083
CT severity score	0.049	0.557	1.050	0.893–1.235
WBC (G/L) at ICU admission	0.001	0.385	1.001	0.999–1.003
CRP (mg/L) at ICU admission	0.003	0.111	1.003	0.999–1.008
IL-6 (pg/ml) at ICU admission	0.001	0.385	1.001	0.999–1.003
Ferritin (ng/ml) at ICU admission	−0.001	0.298	0.999	0.998–1.000
LDH (U/L) at ICU admission	0.001	0.502	1.001	0.999–1.002
WBC (G/L) discharge/death	−0.016	0.600	0.984	0.928–1.044
CRP (mg/L) discharge/death	0.008	0.002	1.008	1.003–1.013
IL-6 (pg/ml) discharge/death	0.001	0.354	1.001	0.999–1.001
Ferritin (ng/ml) discharge/death	0.001	0.001	1.001	1.000–1.002
LDH (U/L) discharge/death	0.001	0.273	1.001	0.999–1.001

CT, computed tomography; ICU; intensive care unit; CRP, C-reactive protein; WBC, White blood cells; IL-6, interleukin-6; LDH, lactate dehydrogenase.

**Table 4:** Multivariate analysis for lethal outcome predictors.

	B	P	HR	CI 0.95%
Age	-	0.066	-	-
Arterial hypertension	-	0.101	-	-
Diabetes mellitus	1.960	<0.001	7.096	3.098–16.253
CRP (mg/L)	0.009	0.022	1.009	1.001–1.016
Ferritin (ng/ml)	0.001	0.013	1.001	1.000–1.002

CRP, C-reactive protein.

## Discussion

This single-center study included 121 patients with COVID-19 who were admitted to the intensive care units (ICU). The study examined the various clinical, laboratory, and microbiological parameters of these patients.

Beneficial use of TZB has been described in many studies and even through prospective open multicenter studies on patients with severe disease (10).

More than half of our patients were male. This finding is consistent with the results of certain meta-analyses that suggest a higher risk of severe COVID-19 among male patients (11). The increased risk for disease severity was higher in older age groups; however, there is no definitive age cut-off point identified. Additionally, older age has been associated with an elevated fatality rate in COVID-19 cases (12). In our study group among the oldest patients (those older than 80 years), a lethal outcome was noticed in 85%. Our data are similar to those reported by the Center for Disease Control and Prevention (CDC), where death occurred in 80%, with the highest percentage being among those older than 85 years (13).

For the follow-up of patients with hyperinflammatory syndrome, it is recommended to monitor CRP (C-reactive protein), ferritin, D-dimer, and LDH (lactate dehydrogenase) in addition to clinical symptoms and radiological findings (14). Multivariate analysis in our study has shown that increased levels of CRP and ferritin after tocilizumab applications in our patients should be considered significant predictors for poor outcomes.

Elevated levels of inflammatory markers such as D-dimer and ferritin, as well as proinflammatory cytokines like IL-6, have been linked to severe COVID-19 disease. Blocking the inflammatory pathways has been hypothesized as a potential strategy to prevent disease progression in these cases (15). Regarding the biomarkers suitable for monitoring tocilizumab (TZB) therapy, the cut-off value for C-reactive protein (CRP) effectiveness differs. Lower CRP and IL-6 values than expected were observed in the studied cohort, likely attributed to the influence of TZB. For instance, in the RECOVERY study, the inclusion criteria required a CRP level greater than 75 mg/L (16), while in the TOCIBRAS and BAC-Bay studies, a CRP level greater than 50 mg/L was used (17, 18). It was shown if CRP was >35 mg/L, TZB reduces mortality up to 35%, while TZB effectiveness in CRP < 35 mg/L was still unknown (19). Regarding CRP findings in our patient group, the significant difference in CRP values after TZB noticed between survivors and non-survivors suggested that increased CRP levels in non-survivors could be due to infections.

We noticed that TZB temporarily increased circulating IL-6 level (because competitive binding with IL-6) receptors, and for this reason, the IL-6 concentration was only recommended at treatment beginning but was not suitable for treatment monitoring (10). Our findings indicated a significant difference in IL-6 levels after tocilizumab (TZB) administration between survivors and non-survivors. This observation may suggest the long half-life of TZB following its application could potentially be attributed to the presence of concomitant superinfection. A recent study reported that critically ill COVID-19 patients exhibited imbalanced iron levels. Hyperferritinemia was also observed in our study and connected with serious lung damage, which increased the susceptibility of COVID-19 patients to superinfections. Due to the lower blood pH and increased levels of pCO2 in COVID-19, ferritin becomes unstable and has a tendency to release iron. This released iron can be readily taken up by various pathogens, potentially leading to infection. Iron deficiency 2 months after COVID-19 disease predisposes recovered patients to high risk of fungal disease (20). Regarding our results ferritin level after TZB administration among non-survivors was significantly higher and indicated that superinfections could be an important risk factor for lethal outcomes. In multivariate analysis, ferritin level was found to be an important predictor of lethal outcome.

Numerous studies have tried to show an adequate time for TZB effectiveness. In the REMAP-CAP study, TZB was effective within 2 days of referral to ICU (8). Similar results were shared by StopCovid investigators, suggesting that patients who received TZB within 2 days of admission to the ICU had a reduced risk of death compared to patients who did not receive TZB. In contrast to our study where all patients (100%) received corticosteroid treatment, a significantly lower percentage of patients (18.5%) were treated with concomitant corticosteroids and Tocilizumab (TZB) in some previous reports (21). In the CHIC study, TZB was administered after initial treatment with corticosteroids but did not show a better response (22). Recovery study demonstrated improved survival outcomes in patients treated with TZB (31 vs. 33% *p* = 0.0028) reduced the need for invasive mechanical ventilation and increased the likelihood of hospital discharge within 28 days (16). Steroids, like dexamethasone and methylprednisolone, have been widely employed to resolve hyperinflammation and inflammatory lung damage in COVID-19 patients. Following the publication of the RECOVERY trial, dexamethasone was approved by the World Health Organization (WHO) as an immunomodulatory drug for use in COVID-19 patients requiring oxygen. The benefit of dexamethasone was particularly notable in patients who required mechanical ventilation. Since the onset of the pandemic, glucocorticoids have been recommended for critically ill COVID-19 patients (23). Methylprednisolone therapy was associated with decreased rate of progression to mechanical ventilation and an increased likelihood of successful extubation in patients requiring mechanical ventilation (24). In our study cohort methylprednisolone was usually used in doses of 2–3 mg/kg and, in severe forms of COVID-19, 5–7 mg/kg.

Results from most successful studies showed that concomitant use of TZB and corticosteroids was more effective than TZB monotherapy (25). The results of our study demonstrated a high mortality rate (82.5%) among critically ill patients with COVID-19 who required mechanical ventilation. These findings correspond with previously published papers that have also reported high mortality rates in this patient population (26–28).

All of our patients required respiratory support at the time of ICU admission. Previously published papers showed that after TZB administration, invasive MV was applied in 29%, CPAP in 42%, and HFNC in 29%; our study results showed MV being applied in 57 (47.1%), CPAP in 13 (10.7%), and HFNC in 26 (21.5%) (8). A large percentage of intubated patients in our study could be explained with severe forms of COVID-19 at hospital admission and rapid clinical deterioration. The average time for worsening after TZB administration was 3 days. Apart from invasive mechanical ventilation upon deterioration, the duration of symptoms prior to hospitalization is an independent predictor of mortality in these patients. Published data from the early stages of the pandemic have indicated death rates ranging up to 62% in patients admitted to the intensive care unit (ICU), while in mechanically ventilated patients, the death rate reached up to 97% (29). The mortality rate among intubated patients with COVID-19 is higher when compared to patients with other viral pneumonia who also require mechanical ventilation (67 vs. 22%) (27, 28). Published results were still controversial; there are concerns ranging from how mechanical ventilation should be avoided in COVID-19 to those regarding the beneficial role of early intubation in COVID-19 patients (30, 31). Our results showed that only 3 days were spent on MV for non-survivals, suggesting critical forms of disease or an inappropriate patient selection for TZB applications. This question could be without answer. According to our results, among survivors, only 5(8.6%) patients were successfully extubated. Some authors showed that the higher mortality rate in critically ill COVID-19 patients is due to older age, the impact of comorbidities, higher D-dimer values, higher CRP, use of invasive mechanical ventilation, vasopressors, and renal replacement therapy. Our results concluded that the presence of comorbidities such as arterial hypertension and diabetes mellitus were as significantly associated with unfavorable outcomes, which was not in consent with findings of some authors (32). The study period coincided with the dominance of the Delta variant of the SARS-CoV-2 virus, and the majority of our patients were infected by this variant. The Delta variant exhibited higher contagiousness with a mortality rate 133% higher than the original strain, while the hospitalization risk rose by 108%, and the probability of ICU admission increased by 235% (33). The comparative analysis of radiological changes between the survivors and non-survivors after TZB administration showed progression of dominantly interstitial lesions in 85 (70.4%) patients without significant difference between survivors and non survivors (75.9 vs. 65.1%). Our results are consistent with previously published data when it comes to non-survivors, but they are not consistent regarding survivors where TZB administration was found to be beneficial (34).

Comorbidities have been consistently linked to severe illness and mortality in patients with SARS-CoV-2 infection. In some previous reported papers, more than two underlying conditions were found as predictors for disease severity as was also shown in our paper (35). In accordance with these data, among the patients included in our study, 94 (77.7%) patients had two or more preexisting comorbidities, which are recognized as potential risk factors for disease severity and lethal outcomes. Survival analysis demonstrated the prognostic significance of arterial hypertension and diabetes mellitus when compared to other clinical and therapeutic variables as predictors for survival. Moreover, the multivariate analysis indicated that diabetes mellitus emerged as the most significant risk factor for lethal outcomes.

In addition to the effect on survival, after TZB use, some authors have noticed large number of superinfections, like pneumonia and bloodstream infections in 39% patients (36). Superinfections in COVID-19 patients requiring transfer to the ICU are recognized as an important and challenging complications. There is a lack of information about superinfections in COVID-19 patients because most of these patients were treated with broad-spectrum antibiotics and antimycotics. Indeed, the use of drug targeting therapy like antagonists of IL-1 and IL-6 receptors has been associated with an increased risk of superinfection in patients with COVID-19 (37). There are recommendations to avoid TZB in patients with immunosuppression, alanine aminotransferase levels greater than five times the upper normal limit, in patients with high risk for gastrointestinal perforation, in serious bacterial or fungal infections, in patients with an absolute neutrophil count <500 cells/μl, platelet count <50.000 cells/μl, or known hypersensitivity (38). In countries highly burdened by tuberculosis, screening for latent tuberculosis is mandatory before TZB use, much like how prophylactic treatment with ivermectin for strongyloidiasis in endemic areas (39). However, current guidelines were not focused on identification of patients with risk of fungal or bacterial infections after tocilizumab administration such as those with preexisting lung diseases. In some cases, post–treatment monitoring of patients and early detection for bacterial and fungal infection is needed (40). Increased infection risk may be compounded with concomitant use of glucocorticoids with TZB (41). Bacterial infections and superinfections after TZB use were seen in various studies. According to Menzella et al., patients who were treated with TZB had a nearly two times higher susceptibility to developing secondary bacterial infections or superinfections compared to the non-treated group (42). The most superinfections found were ventilator-associated pneumonia caused by *Staphylococcus aureus* in about 50% of cases. In the literature, there have been reports of candidemia and invasive pulmonary aspergillosis occurring in patients who were treated with TZB (43, 44). In our study cohort, bacteriemia was found in 20 (16.53%) patients, with *Acinetobacter* spp. being most frequently found, as was described in some previous papers. It is known that prolonged stays in the intensive care unit (ICU) for COVID-19 patients and the use of immunomodulatory therapies, including TZB, can potentially elevate the risk of superinfections caused by multidrug-resistant *Acinetobacter* spp., which presents one of the most dangerous threats to life (45). Bacteriemia after TZB administration in our study was a significant cause of death in comparison with patients with sterile blood cultures (*p* = 0.031) (36). In our study group, urosepsis was found to be a significant predictor for lethal outcome, in 9 (7.4%) patients, with *Candida* spp. being most frequently isolated (*p* = 0.003). The survival rate according to bacteriemia and urosepsis was significantly lower. We assume that number of isolates from blood, central venous catheters, or urine cultures should be greater because, at time of admission to ICU, our patients were treated with broad-spectrum antibiotics, namely cephalosporins of the second or third generation, fluorochinolones, and carbapemens (46). Blocking the IL-6 pathway is crucial for immune system function and may contribute to infections and superinfections due to immune system suppression. IL-6 plays role as differentiation factor for B cells to synthesize immunoglobulins. IL-6 plays a vital role in antibody synthesis, and drugs like TZB may impact immune defense against COVID-19 by affecting IL-6-mediated antibody production (47). Contrary to some reports, certain authors have observed that the administration of TZB in COVID-19 patients did not result in a decreased antibody response to SARS-CoV-2 (47, 48).

Tocilizumab’s place in the treatment of COVID-19 is not clearly defined. There are many controversial dilemmas about TZB use in COVID-19, like TZB monotherapy versus combinations with corticosteroids, the timing of TZB administration, patient selection, as well as a cost–benefit analysis of TZB therapy (49).

Likewise, the results of several studies failed to prove the benefits of TZB because inclusion criteria were based on a lower degree of respiratory insufficiency with oxygen support up to 10 liters or high flow nasal cannula with small number of intubated patients (9).

Our study has few limitations. First of all, data of concomitant corticosteroids use and the inclusion criteria for TZB applications in prior hospitals were unknown. Our study group lacked a control group for comparison, precluding patients with the same condition who did not receive TZB. Thoracic computed tomography was done at the time of admission but not during treatment for the majority of our patients.

## Conclusion

In our study, despite the current guidelines recommending the use of Tocilizumab in patients with severe forms of COVID-19, we did not find any evidence of its beneficial effects. The high mortality rate among intubated patients after Tocilizumab use suggests appropriate patient selection and monitoring. Clinicians should keep in mind the risk of superinfections after Tocilizumab administration. The concomitant use of corticosteroids and structural lung damage seen in COVID-19 pneumonia may potentially increase the risk of infection. Diabetes mellitus as well as increased levels of CRP and ferritin were found to be the strongest predictors for a poor outcome, and this was not the case for increased levels of IL-6 as was mentioned before. Authors of this manuscript strongly believe that Tocilizumab effectiveness varies between different waves of SARS-CoV 2. Indeed, randomized controlled trials will be necessary for further investigations into the effectiveness of Tocilizumab in COVID-19 patients.

## Data availability statement

The raw data supporting the conclusions of this article will be made available by the authors, without undue reservation.

## Ethics statement

The studies involving humans were approved by Ethics Committee of the Clinical Center of Serbia. The studies were conducted in accordance with the local legislation and institutional requirements. Written informed consent for participation was not required from the participants or the participants’ legal guardians/next of kin because the majority of patients were in a critical clinical condition, on mechanical ventilation, hence unable to provide written consent. Additionally, the study was observational, and the principle of patient anonymity was observed.

## Author contributions

VB and TA-V contributed to conception and design of the study. AR organized the database. VB performed the statistical analysis. TA-V wrote the first draft of the manuscript. DM wrote sections of the manuscript. All authors contributed to the article and approved the submitted version.
